# The Maternal Blood Transcriptome Reflects Changes in Fetal Growth and Is an Accurate Predictor of Birth Weight in Cattle

**DOI:** 10.1096/fj.202502648R

**Published:** 2025-11-04

**Authors:** Laura Thompson, Pim G. van Helvoort, Maurice Duijn, Daphne D. Reinders, Eliza M. Murphy, Michael McDonald, Alan D. Crowe, Stephen T. Butler, Patrick Lonergan, Maria Belen Rabaglino

**Affiliations:** ^1^ School of Agriculture and Food Science University College Dublin Dublin Ireland; ^2^ Department of Population Health Sciences, Faculty of Veterinary Medicine Utrecht University Utrecht The Netherlands; ^3^ Teagasc Animal and Grassland Research and Innovation Centre, Moorepark, Fermoy, Co. Cork Ireland

**Keywords:** biomarkers, gene expression profiling, gestation, machine learning, transcriptomics

## Abstract

Harnessing information from maternal blood to predict fetal growth is an emerging area of research in livestock production, offering a noninvasive tool to monitor development. This study aimed to investigate temporal changes in blood gene expression during cow gestation through a bioinformatic approach and to determine the association between transcriptomic modifications in maternal blood at day 63 of gestation and calf birth weight (BW). Publicly available gene datasets from gestational days 0, 21, 42, 56, 63, and 105 were integrated to investigate maternal gene expression changes across gestation. Coexpression clustering identified four clusters, with three enriched for relevant biological processes, including interferon response genes from day 0 to 21, oxidative phosphorylation at day 42, and immune response genes by day 105. Additionally, maternal blood samples from 20 recipient cows carrying female fetuses derived from in vitro‐produced embryos were subjected to RNA sequencing. Unsupervised weighted gene co‐expression network analysis of these data identified four modules of coexpressed genes correlated with calf BW (*p* < 0.05). Supervised analysis revealed 189 differentially expressed genes (DEG; FDR < 0.05) associated with calf BW. The 106 positively associated DEG enriched phosphorylation and protein modification, while the 83 negatively associated DEG enriched immune processes. Biomarker genes were best determined using genes affected by gestational age and DEG, yielding 26 and 17 biomarkers, respectively, with *R* ^2^ values of 0.88 and 0.75 for predicting calf BW. In conclusion, transcriptomic analysis of the maternal blood identified biomarkers associated with calf BW and may have application in other species.

## Graphical abstract

A bioinformatics approach demonstrated that the maternal blood transcriptome changes throughout gestation, with coexpression clustering indicating interferon responses early on, oxidative phosphorylation mid‐gestation, and immune pathways later in pregnancy. On day 63 of gestation, bioinformatics analyses identified biomarker genes in maternal blood whose expression levels accurately predicted calf birth weight, which could be used to develop an early tool for estimating offspring birth weight. Our results support the role of maternal blood as a sensor of fetal growth in cattle.

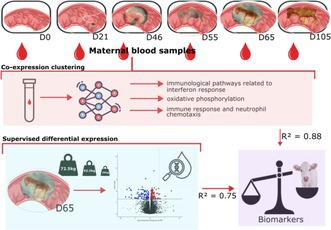

## 1 Introduction

Pregnancy in mammals is a complex and finely orchestrated biological process. A successful pregnancy involves coordinated communication between a partially (semi‐allogenic) or entirely (allogenic) genetically foreign embryo and the maternal environment. If embryo implantation succeeds, the fetal period involves regulated growth of the fetus and organ development to prepare the individual for postnatal life. During that time, the maternal body undergoes pronounced metabolic and endocrine adaptations, impacting the physiology of all maternal organs. In particular, the maternal immune system experiences profound changes to tolerate the growth of the semi‐allogenic fetus [1]. These adaptations trigger a response in circulating blood cells that is evidenced through alterations in the peripheral blood transcriptome, which is contributed to by both maternal blood cells and cell‐free fetal RNA [2]. Humans exhibit invasive, haemochorial, placentation, in which trophoblast cells (placenta precursors) come into direct contact with maternal blood, resulting in the presence of cell‐free fetal RNA in maternal circulation [3]. Furthermore, the maternal blood transcriptomic profile has been used to predict gestational age [4], risk of preterm birth [4], and even birth weight (BW) through samples obtained at the beginning of the third trimester [5]. The impact of pregnancy on the maternal blood transcriptome in species with a more superficial implantation, and whether it is possible to harness the maternal blood transcriptome during early gestation in such species to predict newborn health, is largely unknown.

In cattle, a species with a synepitheliochorial placentation, that is, with additional tissue layers in the interhaemal membrane, the maternal blood transcriptome appears to reflect gestational changes. For instance, the maternal peripheral white blood cell transcriptome already differs at day 21 postovulation between pregnant and nonpregnant cows [6]; indeed, expression of interferon‐stimulated genes in blood cells is used as an early tool for pregnancy diagnosis [7]. Furthermore, oversized fetuses are associated with changes in the maternal blood transcriptome at 55 and 105 days of gestation [8], and notably, we were able to demonstrate a molecular signature in the maternal blood transcriptome that predicts fetal weight during the first trimester of gestation with very low error [9]. It is still unknown, however, whether there are gene transcripts in maternal blood that exhibit temporal dynamics in expression across gestation. Even more intriguing, a molecular signature in the maternal blood may be correlated with characteristics of the newborn, even when the fetus is fully allogenic (e.g., after the transfer of an embryo derived from a sire and dam unrelated to the recipient, as is the norm in commercial embryo transfer in cattle).

Bovine species represent an excellent model for studying the outcomes of assisted reproductive technologies (ART) since embryo and fetal development in humans and the cow share more similarity than in humans and the mouse [10]. In cattle, large offspring syndrome (LOS) is a condition associated with calves derived from in vitro‐produced (IVP) embryos, and is characterized by excessive fetal growth, prolonged gestation, increased incidence of dystocia, and greater risk of perinatal mortality [11, 12]. Given that LOS has been linked to placental dysfunction and abnormal fetal–maternal interactions, studying the maternal blood transcriptome in pregnancies derived from IVP embryos may provide insights into early molecular signatures associated with fetal overgrowth. Therefore, the objectives of this study were (i) to apply a bioinformatic approach to determine temporal changes in blood gene expression during cow gestation and (ii) to apply unsupervised and supervised bioinformatic approaches to detect a transcriptomic signature in maternal blood at approximately 2 months of gestation that is predictive of calf BW. We hypothesized that the molecular profile of circulating blood cells in cattle is influenced by, and reflective of, fetal development. Since fetal organs are already differentiated around 65 days of gestation [13], the accompanying maternal blood transcriptome could provide an accurate snapshot of fetal size and even predict calf BW if the fetus continues a proportional growth trajectory. Therefore, molecular signatures in maternal blood can be used to predict fetal and postnatal features of the offspring.

## 2 Materials and Methods

### 2.1 Determination of Gestational Changes in the Maternal Blood Transcriptome Through a Bioinformatic Approach

Integration of blood transcriptomic data at different gestational ages was implemented using R packages. Data were obtained from three published studies (GSE210665 [6], GSE230294 [9], GSE179946 [8]) plus the data from the study detailed below, spanning gestational ages from 0, 21, 42, 55, 63, and 105 days. In all studies, pregnancies were diagnosed after embryo transfer, although for the GSE179946 dataset, pregnancies were also achieved through artificial insemination. However, only the samples from embryo transfer pregnancies were analyzed. More detailed characteristics about each study are presented in Table 1. Batch (i.e., individual study) effects were removed with ComBat‐seq from the sva package [14], and RUVg from the RUVSeq package was used for normalization [15]. This latter method uses negative control genes to remove unwanted variation. Thus, after removing the batch effects, transcriptome data from pregnant and nonpregnant dams were compared to determine negative control genes with the DESeq2 package [16], which were defined as those overlapping genes with a Benjamini‐Hochberg false discovery rate (FDR) > 0.99 and a log2 fold change < 0.01. The integrated and normalized data were subjected to a time course gene expression analysis using maSigPro package [17]. Genes were fitted using a quintic polynomial regression, and temporally differentially expressed genes (DEG) were identified after FDR correction (*Q* < 0.05). Thereafter, stepwise regression was used to retain the DEG with *R*‐squared > 0.5, which were grouped into four clusters according to similarity in expression profiles through hierarchical clustering. An overrepresentation analysis of the genes within each cluster was conducted using ShinyGO V0.741 [18] to determine enriched ontological terms (FDR < 0.05).

**TABLE 1:** Characteristics of each study used to integrate the corresponding blood transcriptome datasets.

GEO accession	Recipient breed	Recipient age	Gestational time point	*N* (samples)
GSE210665	Holstein	1–2 years	Day 0	5
			Day 21	5
GSE230294	Limousin, Charolais, or Aberdeen Angus cross	2 years	Day 42	10
GSE179946	Angus crossbred	2 years	Day 55	11
			Day 105	11
GSE303819	Angus	1–9 years	Day 63	20

### 2.2 Identification of a Molecular Signature in the Maternal Blood at 63 Days of Gestation Predictive of Calf Birth Weight

#### 2.2.1 Animals

All experimental procedures involving animals were approved by the Teagasc Animal Ethics Committee and authorized by the Health Products Regulatory Authority in Ireland, in accordance with Statutory Instrument No. 543 of 2012 under European Union legislation (Directive 2010/63/EU) for the protection of animals used for scientific purposes.

#### 2.2.2 Oocyte Production, Embryo Transfer, and Fetal Measurements

Details regarding the generation of the pregnancies can be found in a previous publication [19]. Briefly, oocytes were collected from Holstein‐Friesian dairy donors (ET‐DAIR; *n* = 51) and Angus beef donors (ET‐BEEF; *n* = 34) via transvaginal ovum pick‐up. These oocytes were subsequently matured and fertilized in vitro using either conventional or X‐sorted semen from a panel of Holstein Friesian and Angus sires. The resulting presumptive zygotes were cultured in vitro, and a single Grade 1 blastocyst was transferred into synchronized lactating dairy cow recipients on day 7 postestrus. Pregnancy was diagnosed at day 35 and confirmed at day 63 of gestation using transrectal ultrasonography. On day 63, fetal sex was determined based on the position of the genital tubercle. In addition, a one‐minute video of each fetus was recorded and later assessed to measure thoracic diameter (TD) and biparietal diameter (BPD) using ImageJ software. Each image was labeled with the recipient tag number, the sex of the fetus, and the values for TD and BPD.

#### 2.2.3 Day 63 Maternal Blood Collection and Processing

To remove confounding effects of breed type and calf sex while ensuring a sufficient range in calf BW, day 63 maternal blood samples for a subset of female calves (*n* = 20) derived from oocytes collected from beef dams fertilized with sex‐sorted semen were used in the current study.

Maternal blood samples (*n* = 20) were collected via tail venipuncture using a 21‐gage vacutainer needle on day 63 before conducting transrectal ultrasonography. The skin around the target area was sanitized with methylated spirits before collection. For stabilization and isolation of total RNA from whole blood for gene expression analysis, blood was drawn directly into Tempus Blood RNA Tubes (ThermoFisher Scientific) and shaken vigorously for 10–20 s to maximize RNA yield. All blood samples were kept at −20°C for 24 h and then moved to −80°C for storage until processing.

#### 2.2.4 Calf Birth Weights and Statistical Analyses

Calf BW was recorded at parturition (*n* = 20). Calving ease was categorized as ‘normal’ (unassisted) or requiring assistance (‘assisted’). Correlations between measurements of fetal size at day 63 (TD and BPD) and calf BW were assessed through the Spearman correlation coefficient in *R*. The effects of fetal size at day 63, dam parity, and calf BW on the odds of requiring assisted calving or not were determined through logistic regression using the glm function for R, selecting the final model through step backward selection.

#### 2.2.5 RNA Extraction, Library Preparation, and RNA Sequencing

Blood samples were shipped on dry ice to Macrogen Europe (Amsterdam, The Netherlands), and RNA extraction, quality control, library preparation, and sequencing were conducted. Libraries were prepared with Truseq‐stranded mRNA for all tissue samples, followed by PE100 sequencing with the NovaSeq platform, generating ~30 million raw reads per sample in FASTQ format. The read qualities for each FASTQ file were assessed with FastQC (http://www.bioinformatics.babraham.ac.uk/projects/fastqc/), and low‐quality bases and adapters were removed with Trimmomatic (V 0.39) 133 [20]. The sequenced reads were mapped to the bovine reference genome (bosTau 9) with the STAR aligner (V 2.7.0b) [21], generating the genome index with the ARS‐UCD1.3 assembly. On average, 87.3% of the reads were uniquely mapped to the genome, ranging from 83.4% to 89.9%. Read counts were estimated at the gene level, and the counting was implemented using featureCounts [22], part of the Subread software (V 2.0.3). Data are deposited in NCBI's Gene Expression Omnibus and are accessible through GEO accession number GSE303819.

### 2.3 Bioinformatic Analysis

#### 2.3.1 Unsupervised Analysis

Genes with zero counts in all samples were filtered out before the analyses, and the expression data were normalized through variance stabilizing transformation [23]. Next, the 25% most variable genes across the samples (5791 genes) were retained to determine clusters of coexpressed genes through Weighted Gene Coexpression Network Analysis (WGCNA) [24]. Clusters or modules of coexpressed genes in the resulting expression dataset were identified through a block‐wise method, which constructed a signed network using biweight midcorrelation and a soft thresholding power (*β*) of 7 to calculate adjacency. Pearson correlations and corresponding student asymptotic *p*‐values were calculated between the modules and the calf BW to identify modules (or clusters) of coexpressed genes that were significantly correlated with calf BW.

#### 2.3.2 Supervised Analysis

DEG associated with calf BW were identified through the DESeq2 [16] package in R. To ensure the biological relevance of subsequently identified biomarkers associated with calf BW, one calf with an extreme BW (72.5 kg, more than two standard deviations greater than the mean BW and thus was considered an outlier) was excluded from the differential expression analysis to reduce potentially confounding noise. By limiting the analysis to the remaining 19 calves with physiologically normal birth weights, variance from this outlier is reduced to better focus on gene expression patterns associated with calf BW. Gene expression counts were normalized by library size with DESeq2 methods, and the normalized count data were modeled as a function of calf BW. The association between gene expression levels and calf BW was tested using the Wald test statistic, adjusting the *p*‐values with Benjamini‐Hochberg method. Genes with FDR < 0.05 were considered DEG, that is, significantly associated with calf BW.

#### 2.3.3 Functional Analysis

Overrepresentation of ontological terms (FDR < 0.05) enriched among the coexpressed genes correlated with calf BW or among the DEG was determined using ShinyGO V0.741 [18].

#### 2.3.4 Comparison Among Studies

The list of genes in the four clusters related to gestational age identified by the bioinformatic approach was compared with the list of coexpressed genes significantly correlated with calf BW and the list of DEG (FDR < 0.1) using Venn Diagrams.

### 2.4 Biomarker Selection

The list of genes used to identify potential biomarkers in the day 63 maternal blood sample for calf BW was defined as follows: (1) ‘Gestational genes’: that is, genes affected by gestational age, as determined through the bioinformatic approach described above (*n* = 1013 genes). (2) ‘Co‐expressed genes’: that is, genes significantly correlated with calf BW, identified using unsupervised WGCNA analysis. (3) DEG, positively or negatively associated with calf BW, determined via supervised analysis. Given the limited sample size, a Monte Carlo cross‐validation (MCCV) strategy was employed. In each of 100 iterations, 13 samples were randomly selected to train a support vector regression (SVR) model with a linear kernel to predict calf BW from blood gene expression, employing leave‐one‐out cross‐validation (LOOCV) as internal validation. The model was implemented with the kernlab package [25], interfaced through the caret package [26]. The expressions of the same genes in the remaining seven samples were used to test the model. To minimize the risk of overfitting, the lists of coexpressed genes and the list of DEG were reidentified within each training set, and only those genes were used in model training. In each run, gene importance was estimated using the varImp function from caret. Candidate biomarkers were defined as genes that ranked within the top 100 in runs where the model achieved a coefficient of determination (*R* ^2^) > 0.75. The predictive performance of these candidate genes was further assessed through a second round of 12 times repeated MCCV (13/7 splits), evaluating the predictive ability in the held‐out samples. *R* ^2^ was estimated for each run as: *R* ^2^ = 1 − ∑(predicted−observed)^2^/∑(observed−mean (observed))^2^, quantifying the proportion of variance in observed values explained by the model predictions. Finally, a permutation test (Y‐randomization) was performed, in which BW values were randomly permuted while gene expression was left unchanged, and the full modeling pipeline was repeated to generate a null distribution of *R* ^2^ values.

## 3 Results

### 3.1 The Maternal Blood Transcriptomic Profile Exhibits Temporal Changes Across Gestation as Determined Through a Bioinformatic Approach

**FIGURE 1:**
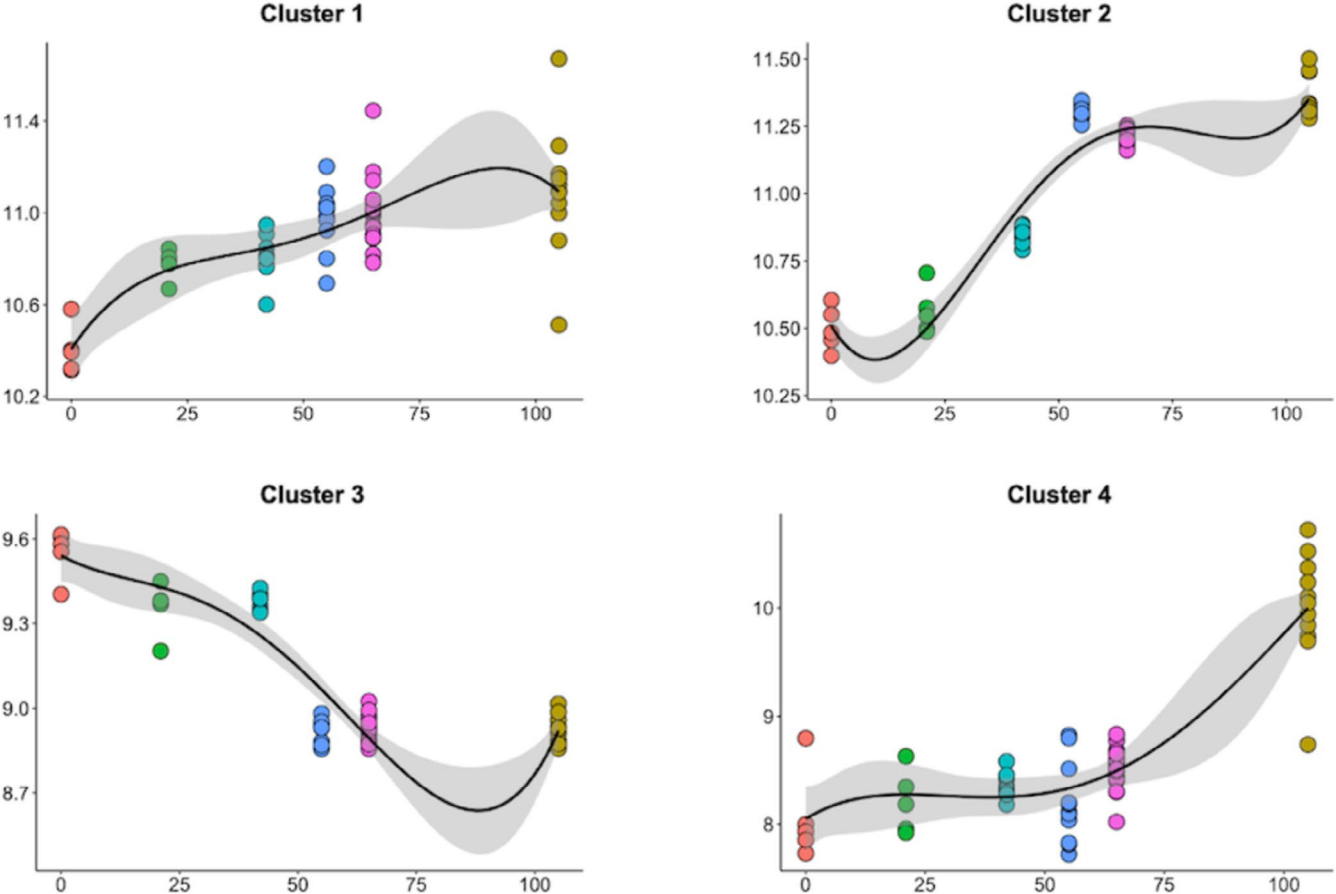
Temporal expression patterns of four gene coexpression clusters in maternal blood across gestation. Each plot represents the average expression trend for one cluster, with samples colored by gestational day: Day 0 (orange), Day 21 (green), Day 42 (light blue), Day 55 (dark blue), Day 63 (pink), and Day 105 (yellow). The number of genes included in each cluster was: Cluster 1 (*n* = 57), Cluster 2 (*n* = 483), Cluster 3 (*n* = 594), and Cluster 4 (*n* = 103).

**FIGURE 2:**
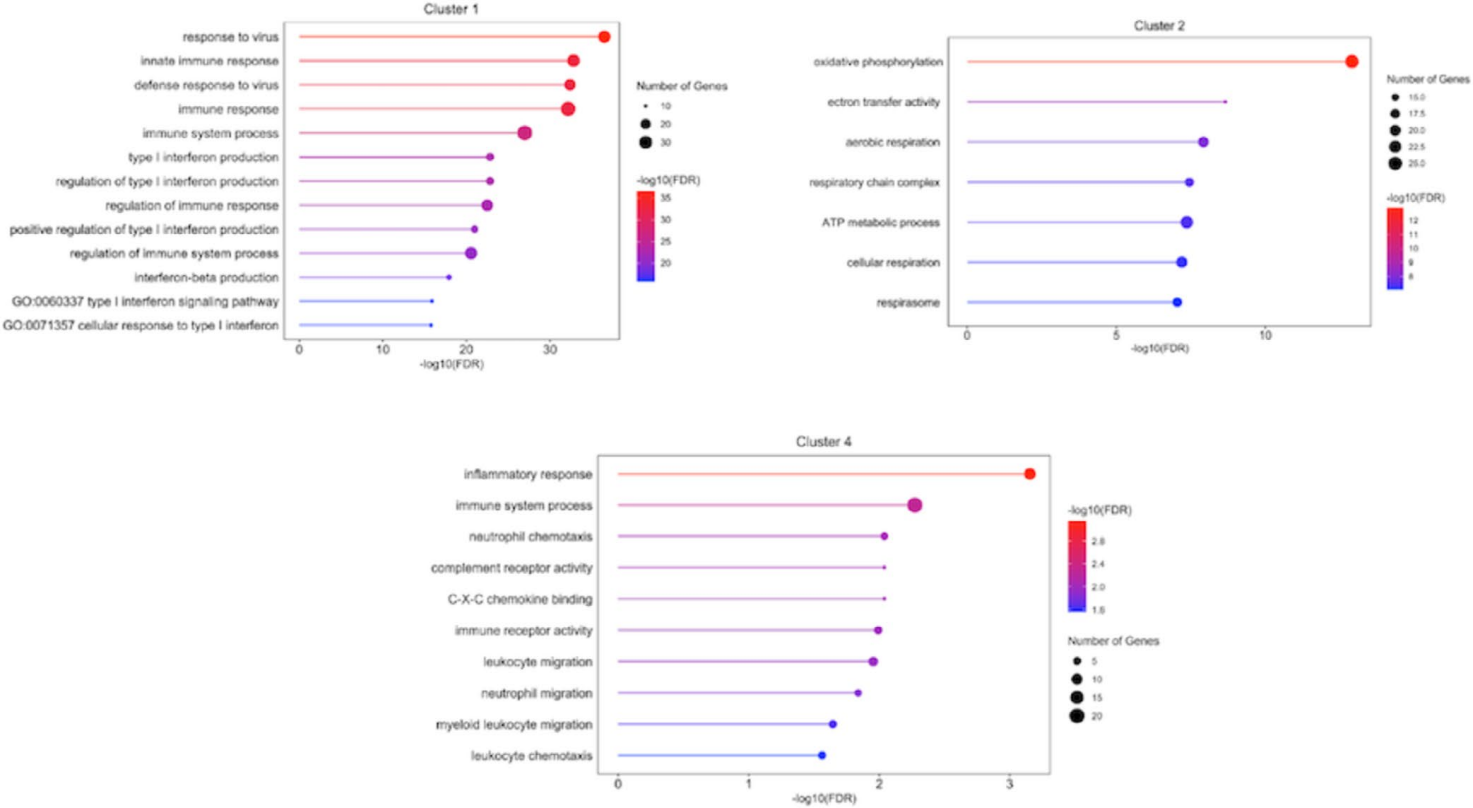
Lollipop plots showing the top enriched ontological terms for Clusters 1, 2, and 4, ordered by –log_10_(FDR), with the number of genes contributing to each term indicated in the legend also. Cluster 1 (*n* = 57) included genes with elevated expression at day 21 (embryonic period), enriching interferon‐related immune pathways. Cluster 2 (*n* = 483) showed increased expression during the fetal period (days 42, 55, and 105) and was enriched for oxidative phosphorylation. Cluster 4 (*n* = 103) was associated with immune response and neutrophil chemotaxis. Cluster 3 (*n* = 594) did not enrich any relevant ontological terms.

Four clusters of coexpressed genes, which had an expression pattern that changed from day 0 to day 105, were identified. These clusters contained 57, 483, 594, and 103 genes, respectively (Figure 1, Table S1). Cluster 1 contained genes that exhibited greater expression at day 21 (embryonic period) compared with day 0 and all the other gestational ages; these genes were involved in immunological pathways related to interferon response. Genes in Cluster 2 exhibited a clear pattern of increased expression during the fetal period (days 42, 55, and 105) compared with days 21 and 0. This gene cluster was strongly enriched for oxidative phosphorylation pathways. Despite having the largest number of genes, Cluster 3 was not significantly enriched for any pathways. Finally, Cluster 4 was enriched for immune response and neutrophil chemotaxis pathways. The main enriched ontological terms for each cluster are indicated in Figure 2, and the entire list is reported in Table S2.

### 3.2 Fetal Morphometrics and Calf Birth Weight

**FIGURE 3:**
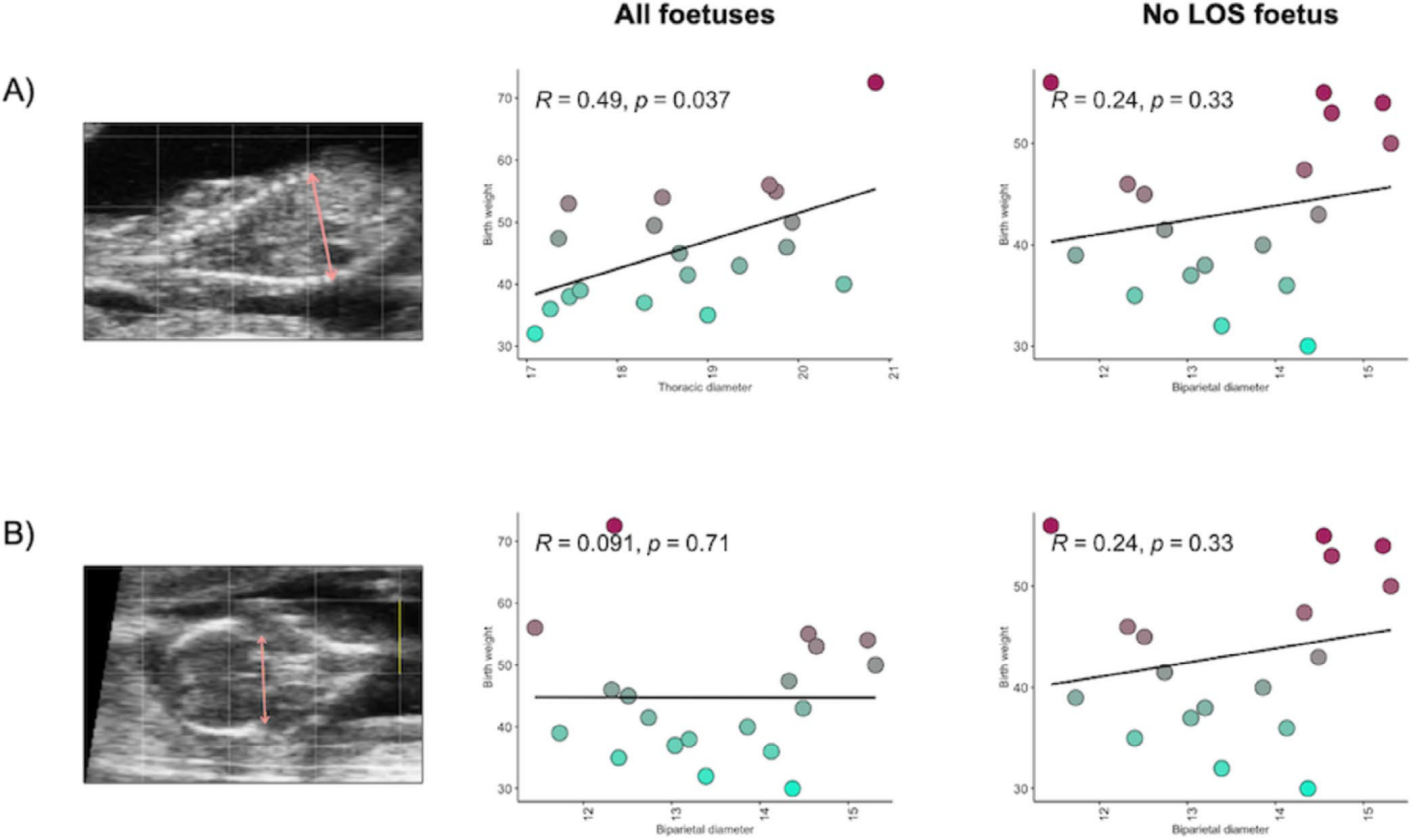
Correlation between thoracic (A) or biparietal (B) diameter (mm) and calf birth weight (BW). Thoracic diameter (TD) showed a moderate positive correlation with calf BW (*R* = 0.49, *p* = 0.035); however, this association was primarily driven by an outlier fetus. When this data point was removed, the correlation between TD and calf BW was no longer significant (*R* = 0.4, *p* = 0.11). No correlation was observed between biparietal diameter and calf BW, either including (*R* = 0.09, *p* = 0.7) or excluding (*R* = 0.24, *p* = 0.3) the outlier fetus.

Measurements for TD and BPD were attempted for all 20 fetuses using the images captured at day 63 of gestation, but one TD and one BPD measurement from two separate calves could not be accurately measured and were therefore excluded. The mean (± standard deviation) TD and BPD were 18.7 ± 1.16 mm and 13.5 ± 1.17 mm, respectively. BPD and TD were not correlated with each other (*R* = −0.11, *p* = 0.66). The mean (± standard deviation) calf BW was 44.9 ± 10.1 kg, with a range from 30.0 to 72.5 kg. Excluding the outlier calf, the mean BW was 43.5 ± 7.7 kg, with a range from 30.0 to 56.0 kg. Thoracic diameter was associated with calf BW (*R* = 0.49, *p* = 0.037), although this association was influenced by the data from the outlier fetus. Removing the data from this fetus removed the correlation between TD and calf BW (*R* = 0.4, *p* = 0.11). BPD was not correlated with calf BW, irrespective of whether the data from this oversized fetus were included (*R* = 0.09, *p* = 0.7) or not (*R* = 0.24, *p* = 0.3) (Figure 3).

Calves that required assistance at delivery had a mean (± standard deviation) BW of 50.6 ± 9.9 kg, whereas calves that had a normal delivery had a mean (± standard deviation) BW of 39.4 ± 5.8 kg. Heavier calves had, on average, 21% higher odds of requiring assisted delivery compared to lighter calves (OR = 1.2 (1.04–1.49)).

### 3.3 Changes in the Maternal Blood Transcriptome Associated With Calf Birth Weight

**FIGURE 4:**
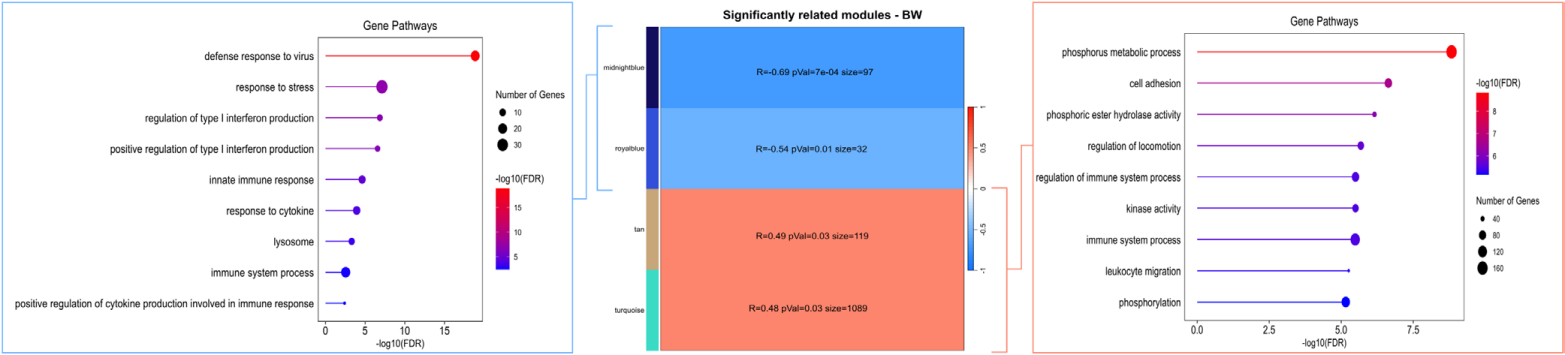
Weighted gene coexpression network analysis (WGCNA) modules correlated with calf birth weight and the functional enrichment of positively and negatively associated modules. Four modules were significantly correlated with calf birth weight: Two positively correlated modules (tan and turquoise) and two negatively correlated modules (midnightblue and royalblue). Lollipop plots show the top enriched ontological terms associated with genes from the negatively correlated modules on the left and positively correlated modules on the right.

**FIGURE 5:**
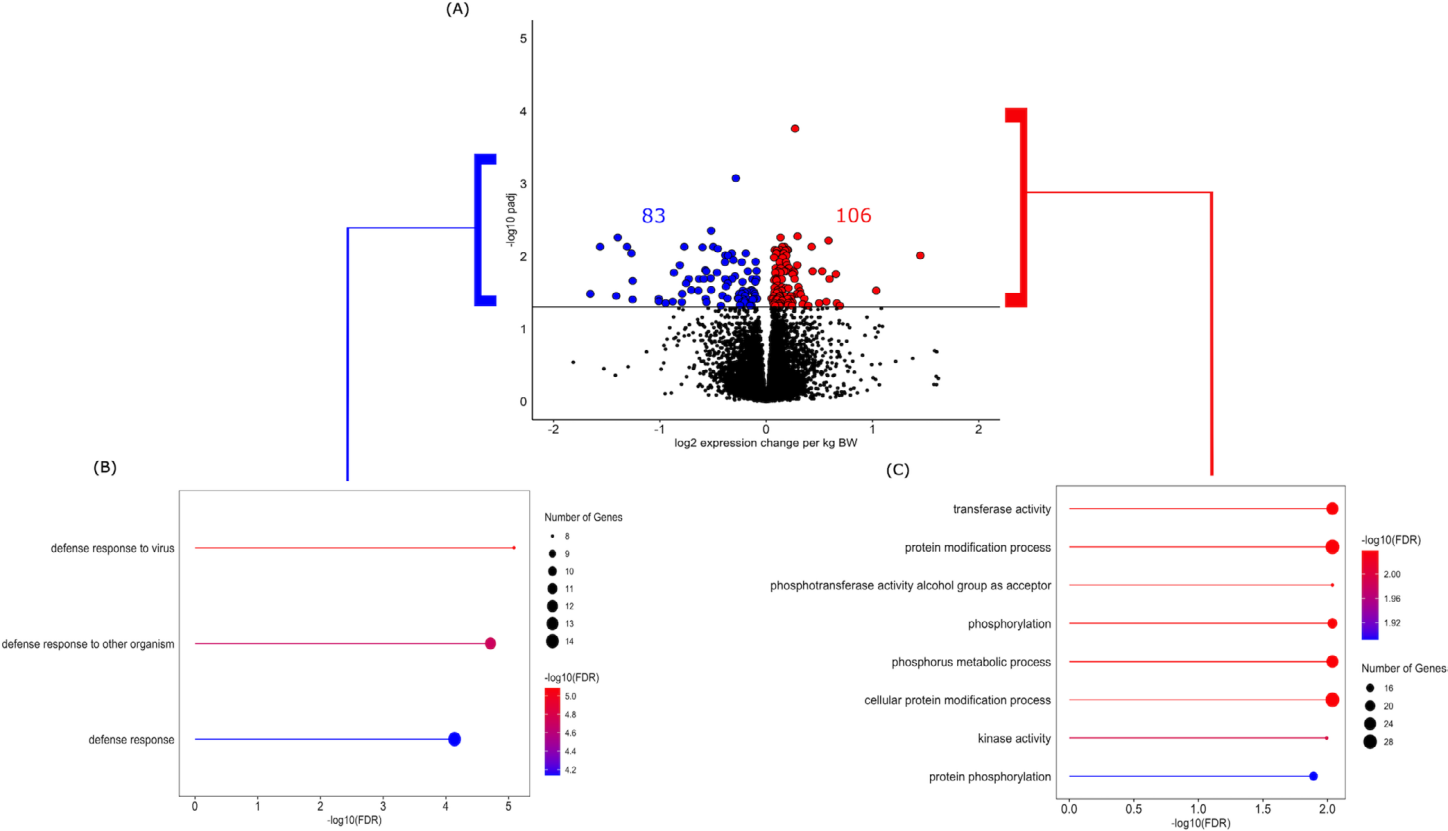
Differential gene expression in the maternal blood at day 63 of gestation, using the subsequent calf birth weight as a continuous variable. (A) Volcano plot depicting differentially expressed genes (DEG) associated with calf BW. Red dots represent genes positively associated with calf birth weight, while blue dots represent genes negatively associated with calf birth weight. (B) Lollipop plot illustrating enrichment analysis for DEG that were negatively associated with calf birth weight. The DEG were interrogated for enriched Gene Ontology terms (Biological Processes) and KEGG pathways using ShinyGO V0.77. The length of the line and the line color correspond to −log10 (FDR), while the size of the dot reflects the number of genes involved in each biological process/pathway. (C) Lollipop plot illustrating enrichment analysis for DEG that were positively associated with calf birth weight.

**FIGURE 6:**
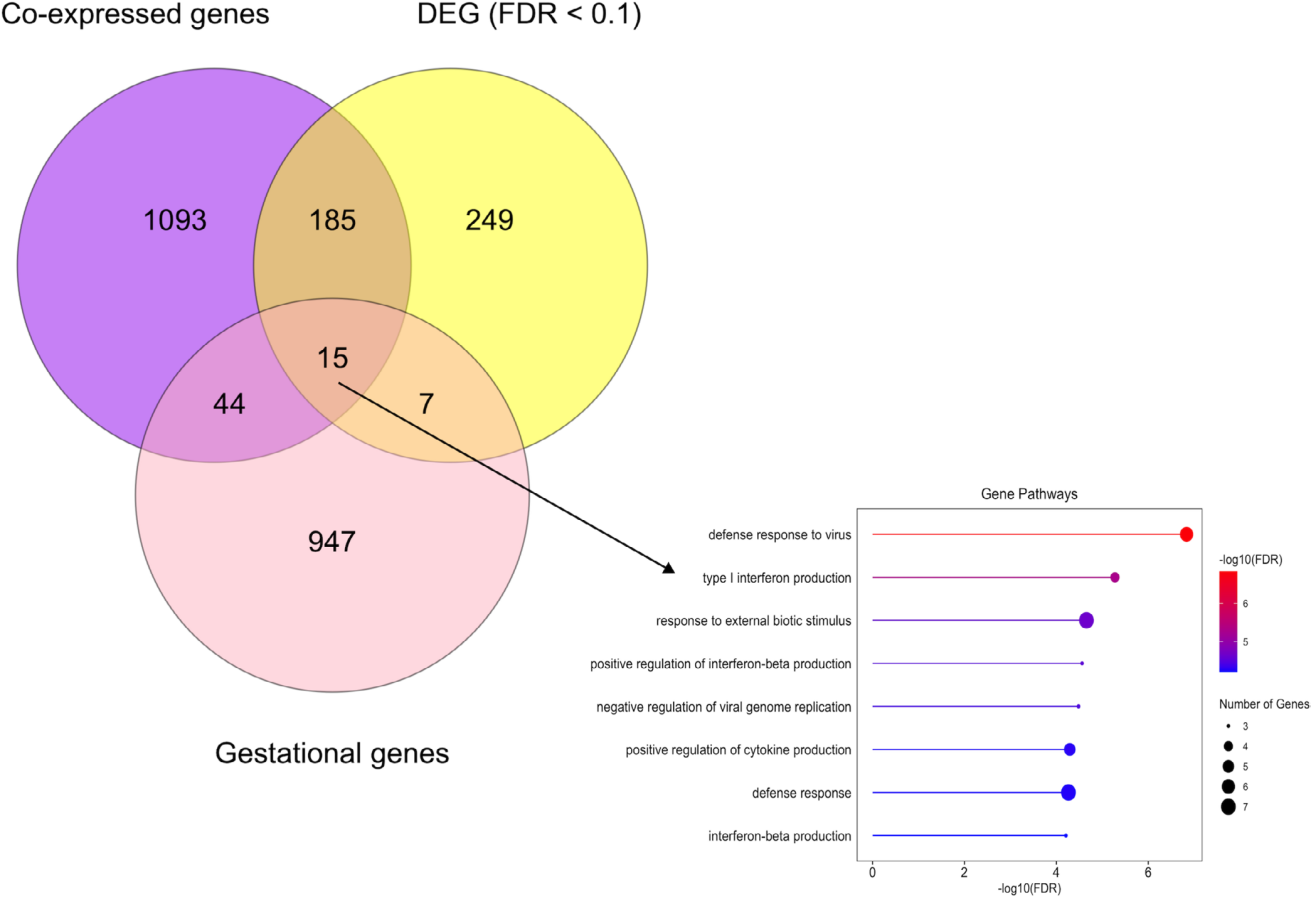
Venn diagram representing the overlap of coexpressed genes identified using WGCNA (purple), DEG (FDR < 0.1) (yellow), and gestational genes (pink).

Application of WGCNA to the recipient blood transcriptome identified four modules of coexpressed genes that were positively (1208 genes, tan and turquoise modules) or negatively (129 genes, midnightblue and royalblue modules) correlated with calf BW (Figure 4, Table S3). Coexpressed genes in the positively correlated modules enriched ontological terms related to phosphorus metabolic process, cell adhesion and regulation of immune system processes. Genes in the negatively correlated modules also enriched immune system processes, as well as response to stress and positive regulation of type 1 interferon production (Table S4). When genes significantly associated with calf BW were identified through a supervised differential expression analysis, 189 DEG (FDR < 0.05) were determined to be significantly associated with calf BW. Of these, 106 DEG were positively associated and 83 DEG were negatively associated with calf BW (Figure 5, Table S5). Genes that were positively associated with BW enriched transferase activity, protein modification processes, and phosphorylation pathways, whereas genes that were negatively associated with BW enriched common processes such as defense response to virus and defense response to other organisms (Table S6). The overlap between the lists of genes obtained from these outputs and the list of genes that changed in expression across gestation is indicated in Figure 6, together with the main ontological terms enriched by those genes.

### 3.4 The Expression of Biomarker Genes in the Maternal Blood Transcriptome are Predictive of Calf Birth Weight

**FIGURE 7:**
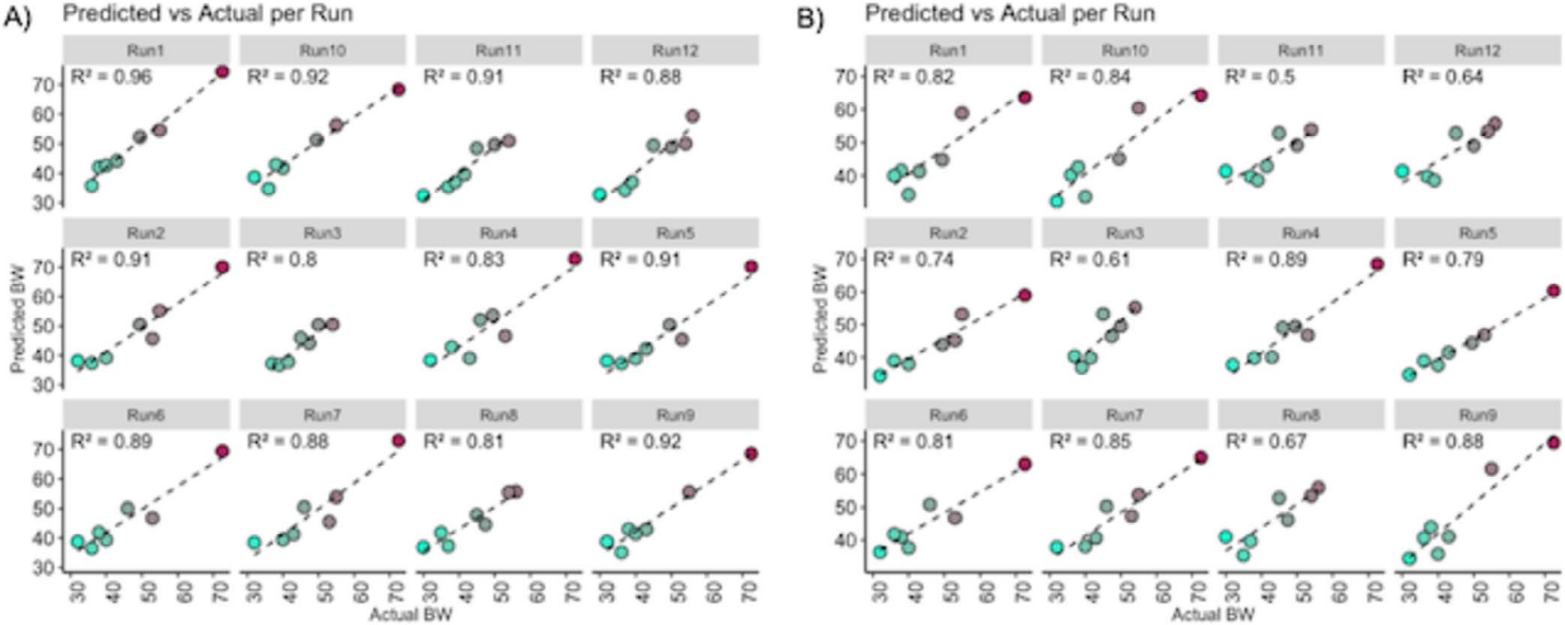
Scatter plots showing the predicted versus actual calf body weights (BW) for seven randomly selected samples. Results from the first nine runs (out of 100 total runs) are displayed. Predictions were based on the expression profiles of genes derived from differentially expressed genes (Panel A) or from genes whose expression changes across gestation (Panel B).

The genes for which the expression was most predictive of calf BW consisted of 26 genes derived from the lists of gestational genes and 17 genes selected from the reidentified DEG within the training set. No candidate biomarker genes were found among the reidentified coexpressed genes. The 26 and 17 candidate biomarker genes achieved overall *R* ^2^ values of 0.88 (0.80 to 0.96) and 0.75 (0.50 to 0.89), respectively, indicating strong predictive power. Conversely, Y‐randomization resulted in predominantly negative *R* ^2^ values (−4.8; −15.6 to 0.5), suggesting that models trained on permuted labels performed worse than simply predicting the mean, supporting the notion that the predictive performances observed with the expression of the 26 and 17 candidate biomarker genes were not due to chance. These gene sets are presented in Table 2, while the average predicted BW values per sample across all runs are reported in Table 3. Figure 7 illustrates the predicted versus actual calf BW for seven randomly selected samples over the 12 runs, based on the expression of the 26 genes (panel A) and 17 genes (panel B). *Interferon regulatory factor 7* (*IRF7*) was a common gene between both lists.

**TABLE 2:** List of biomarker genes identified from the (A) gestational genes list (26 biomarkers) or (B) differentially expressed genes (17 biomarkers).

(A)	
Entrez ID	Name
506138	Apolipoprotein O like (APOOL)
509609	CD101 molecule (CD101)
510073	CD7 molecule (CD7)
538392	CDC42 binding protein kinase alpha (CDC42BPA)
617897	Family with sequence similarity 167 member B (FAM167B)
541255	Interactor of little elongation complex ELL subunit 2 (ICE2)
100125591	Interferon regulatory factor 7 (IRF7)
511387	Kelch like family member 20 (KLHL20)
539267	Lines homolog 1 (LINS1)
507845	Microtubule associated protein RP/EB family member 1 (MAPRE1)
781257	Mitochondrial carrier 1 (MTCH1)
505728	Mitochondrial ribosomal protein L53 (MRPL53)
3283878	NADH dehydrogenase subunit 2 (ND2)
319086	Oncostatin M (OSM)
540457	RAN, member RAS oncogene family (RAN)
506509	SEH1 like nucleoporin (SEH1L)
507828	Serine and arginine rich splicing factor 6 (SRSF6)
509802	Small nuclear ribonucleoprotein polypeptide A (SNRPA)
535727	Solute carrier family 27 member 2 (SLC27A2)
282082	Structure specific recognition protein 1 (SSRP1)
533141	TANK binding kinase 1 (TBK1)
513640	tet methylcytosine dioxygenase 1 (TET1)
281530	Thrombospondin 1 (THBS1)
516516	tRNA methyltransferase 12 homolog (TRMT12)
512912	tubulin gamma complex associated protein 5 (TUBGCP5)
514418	Zinc finger protein 526 (ZNF526)

(B)	
Entrez ID	Name
508458	Acetylserotonin O‐methyltransferase like (ASMTL)
100141457	Beta‐defensin 10 (DEFB10)
523661	Catenin delta 2 (CTNND2)
767985	CDC28 protein kinase regulatory subunit 2 (CKS2)
520143	E2F associated phosphoprotein (EAPP)
532015	ELOVL fatty acid elongase 4 (ELOVL4)
516949	Guanylate binding protein 5 (GBP5)
100125591	Interferon regulatory factor 7 (IRF7)
280846	Lactotransferrin (LTF)
533735	MIB E3 ubiquitin protein ligase 1 (MIB1)
616677	Microtubule associated scaffold protein 2 (MTUS2)
539533	NEDD4 binding protein 2 (N4BP2)
523418	Solute carrier family 38 member 11 (SLC38A11)
614554	TBC1 domain family member 5 (TBC1D5)
524925	TTK protein kinase (TTK)
509471	Ubiquitin conjugating enzyme E2 L6 (UBE2L6)
112446704	Uncharacterized LOC112446704 (LOC112446704)

**TABLE 3:** Average predicted calf birth body weight per sample based on the expression of biomarker genes in maternal blood at 63 days of gestation.

Sample	Actual calf BW	26 biomarker genes			17 biomarker genes		
		Mean predicted calf BW	Residual	Root mean squared error	Mean predicted calf BW	Residual	Root mean squared error
ID_2320	30	34.04	4.04	4.50	41.18	11.18	11.18
ID_9289	32	38.41	6.41	6.41	35.38	3.38	3.88
ID_726	35	41.72	6.72	6.72	35.32	0.32	0.32
ID_1985	36	36.12	0.12	1.00	40.08	4.08	4.19
ID_1625	37	36.03	−0.97	1.58	39.85	2.85	2.87
ID_2266	38	42.52	4.52	4.54	41.73	3.73	3.98
ID_3639	39	36.94	−2.06	2.06	38.01	−0.99	1.22
ID_2130	40	40.37	0.37	1.46	36.41	−3.59	3.99
ID_2686	41.5	38.71	−2.79	2.96	41.37	−0.13	1.46
ID_2734	43	41.90	−1.10	2.04	40.90	−2.10	2.15
ID_1284	45	47.91	2.91	3.18	52.84	7.84	7.84
ID_1273	46	50.78	4.78	4.87	49.99	3.99	4.06
ID_3418	47.4	44.35	−3.05	3.06	46.26	−1.14	1.16
ID_3975	49.5	51.57	2.07	2.41	45.51	−3.99	4.45
ID_9609	50	49.60	−0.40	0.75	49.21	−0.79	0.82
ID_1475	53	45.95	−7.05	7.07	46.57	−6.43	6.47
ID_728	54	51.69	−2.31	3.16	53.91	−0.09	0.79
ID_55	55	55.05	0.05	0.86	57.52	2.52	4.30
ID_1039	56	57.48	1.48	2.39	55.73	−0.27	0.29
ID_298	72.5	70.76	−1.74	2.75	64.14	−8.36	9.01

**FIGURE 8:**
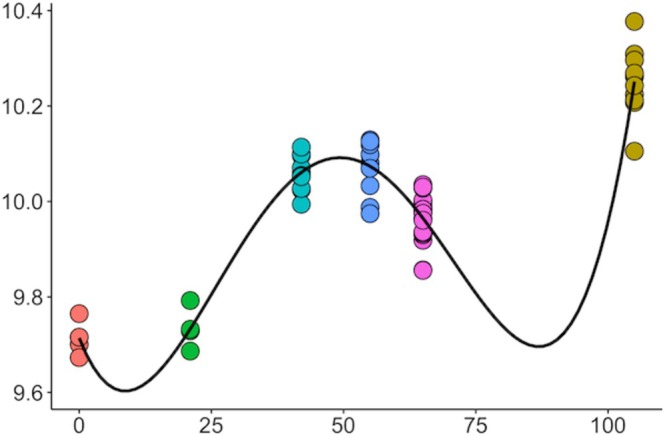
Expression pattern across gestation of the 26 biomarker genes identified as predictors of calf birth weight, selected from the full set of 1013 genes whose expression changes across gestation.

Biomarker discovery from the coexpressed genes identified through WGCNA did not produce results, possibly because WGCNA is designed to detect clusters of coexpressed genes (modules) that correlate with BW at the module level (via eigengenes) and capture broad biological processes, rather than effects at the single‐gene level. Conversely, while the 17 biomarker genes were identified from genes statistically associated with calf BW in the 13 samples used for model training in each run, the 26 biomarker genes were derived from genes whose expression changed with gestational age, based on data pooled from four different studies. Interestingly, the expression of these genes clustered closely by gestational age (Figure 8), which may reflect fetal developmental progression and serve as reliable predictors of calf BW.

## 4 Discussion

In cattle, previous studies reported that the blood transcriptome is altered during very early stages of pregnancy [6, 27]. Whether these changes occur across gestation and potentially can reflect fetal growth is still unknown. This study applied a bioinformatic approach to determine changes in gene expression during cow gestation using publicly available RNA sequencing data from multiple studies, including the current one, and determined the associations between maternal blood gene expression and calf BW. The current study had two main findings: (i) changes in the maternal blood transcriptomic profile across gestation are associated with fetal growth; and (ii) a transcriptomic signature in maternal blood on day 63 of gestation is predictive of calf BW.

During pregnancy, the maternal immune system must undergo profound changes to tolerate fetal growth, triggering an adaptation response in circulating blood cells. In humans, the invasive implantation of the conceptus (around days 8–10 of gestation) [28] and direct contact between maternal blood and the fetal chorionic epithelium of the haemochorial placenta [29] allow pregnancy‐induced alterations to be monitored through the peripheral blood transcriptome, which is comprised of RNA from maternal blood cells and cell‐free fetal RNA [30]. In contrast, there are limited reports of cell‐free fetal RNA or DNA in species with superficial placentation, such as ruminants. Detection of circulating fetal RNA/DNA in pregnant cows is challenging because the synepitheliochorial placenta prevents direct contact between the trophoblast and the maternal blood [31], limiting direct maternal–fetal exchange. In cattle, expression of interferon‐stimulated genes in maternal peripheral blood can be used to determine pregnancy status around the time of maternal recognition of pregnancy [32, 33, 34]. In addition, pregnancy‐associated glycoproteins (PAGs) are produced by trophoblast giant cells; these cells migrate to the endometrial epithelium and secrete PAGs into the maternal circulation starting on d 19 of pregnancy [35], and can be used to estimate the timing of presumptive conceptus attachment [36, 37, 38]. Whether changes in maternal blood induced by the conceptus through either interferon‐tau or PAGs have any association with fetal growth remains largely unknown.

The maternal adaptations to support pregnancy are driven by endocrine and immune signals from the developing conceptus and placenta [39], leading to measurable changes in maternal blood gene expression. Previous studies reported that after embryo transfer, pregnant and nonpregnant recipients exhibited a different physiological status as early as 7 days after transfer, particularly relating to immune tolerance assessed by gene expression changes in peripheral blood cells [6, 27]. This suggests trophoblast signaling and embryo/fetal‐maternal crosstalk are potentially influencing gene expression in maternal circulation, strengthening the ability of the maternal blood to act as a predictor of pregnancy status.

The synepitheliochorial placenta in the cow creates a structural limitation for fetal‐maternal interaction. Despite this, studies in both humans and cows suggest that the maternal blood transcriptome can still capture fetal and placental signals. In humans, fetal‐derived components in maternal circulation reflect fetal status, with maternal blood transcriptomics providing reasonable predictions of BW [5, 40]. Similarly, in cattle, transcriptomic analysis has revealed correlations between maternal blood gene expression and pregnancy status and fetal sex despite the lack of direct blood exchange [6, 41, 42, 43, 44, 45].

Furthermore, coexpressed genes in the maternal blood—the expression levels of which are significantly correlated with those in the fetal heart—have been identified as being associated with fetal weight [9]. This is important, as pregnancies derived from IVP embryos have been associated with a low frequency but unpredictable incidence of an overgrowth phenotype resulting in the birth of large/abnormal offspring [12, 46, 47]. Large offspring syndrome has been reported to be associated with transcriptomic and epigenetic changes induced by suboptimal in vitro culture conditions for embryos [11, 48]. Despite the increase in the number of IVP embryos transferred commercially in recent years [49], follow‐up data on birth outcomes are lacking, and accurate tests to identify LOS pregnancies during gestation do not exist. Strategies to identify LOS pregnancies in utero (preterm) are desirable to facilitate appropriate decision‐making and management during the final weeks of pregnancy and during parturition. Rabaglino et al. [9] recently successfully constructed a predictive model using 35 fetal cardiac genes that were significantly coexpressed with genes in the maternal blood, allowing accurate prediction of fetal weight with low error. It has also been reported that LOS fetuses induce changes in the maternal blood transcriptome at Day 55 and 105 of gestation [8]. This provides further evidence that the maternal blood transcriptome likely captures information on some important factors related to metabolic state and immune status that are associated with fetal growth.

Building on these findings, we integrated transcriptomic data from multiple studies to investigate the temporal changes in maternal blood gene expression across gestation. Four clusters captured the temporal changes in the maternal blood transcriptome across gestation, highlighting the changing relationship as pregnancy progresses. Of these clusters, three enriched relevant ontological terms. Cluster 1 (*n* = 57) included genes with elevated expression at day 21 (embryonic period) and was enriched for interferon‐related immune pathways. Interferon‐tau is recognized as the maternal recognition of pregnancy factor in cattle [50], which is secreted by the conceptus between days 16–19 of gestation in the cow. The expression of interferon‐tau increases as the conceptus undergoes elongation [51], which is critical for pregnancy maintenance. Cluster 2 (*n* = 483) genes exhibited increased expression during the fetal period (days 42, 55, and 105) and were enriched for oxidative phosphorylation pathways. This may reflect enhanced mitochondrial activity during this time in response to fetal growth demands. Rabaglino et al. [9] suggested that as fetal weight was increasing, the molecular profile of each organ indicated greater maturation, which likely requires more energy production by fetal cells that can be sourced from maternal blood. Supporting this, a study in mice reported that as organs matured, genes involved in energy‐related processes such as oxidative phosphorylation and the electron transport chain also became more active [52]. Cluster 4 (*n* = 103) was associated with immune response and neutrophil chemotaxis pathways at day 105 of gestation. The enrichment of these pathways may reflect the immune adaptation that occurs during pregnancy to support fetal development. In humans, changes in immunological state are well recognized throughout pregnancy, with the second phase resembling an anti‐inflammatory phase, where a shift in cytokine bias towards T helper 2 phenotype is thought to contribute to fetal growth [53]. Cattle also undergo immune modulation during gestation, but there is evidence to suggest the enriched immune pathways in maternal blood could be influenced by signals originating from the conceptus [54].

Results from this initial analysis showed that the molecular profile of maternal blood is dynamic and responds to the growing fetus, even when derived from surrogate recipients of allogenic fetuses. Next, considering that the fetus is fully differentiated by about 60 days of pregnancy and continues to grow in relative proportion during gestation, we tested whether the maternal blood transcriptome at this stage can reflect the calf's BW, using both unsupervised (WGCNA) and supervised (DEG) methods to identify blood genes associated with calf BW. Finally, we explored different strategies to identify biomarker genes in the maternal blood at 2 months of gestation whose expression can predict calf BW.

Weighted Gene Coexpression Network Analysis identified four modules associated with calf BW, comprising 1208 positively correlated genes and 129 negatively correlated genes. Positively correlated modules were enriched for biological processes such as phosphorus metabolism and immune regulation, whereas negatively associated modules were enriched for pathways associated with response to stress, positive regulation of type 1 interferon production, and immune system processes. This suggests the immune system may be distinct in dams bearing lighter and heavier calves. One study reported that heavier calves exhibited lower IgG concentration than lighter calves within the first 24 h after birth [55]. This may suggest a trade‐off between fetal growth and immune function in early life. In parallel, supervised differential expression analysis identified 189 DEG significantly associated with calf BW, with positively associated DEG enriching phosphorylation and protein modification, and negatively associated DEG enriching immune processes. This further supports the idea that enhanced fetal growth may occur at the expense of immune development.

Transcriptomic analysis identified 26 and 17 biomarker genes from the gestational genes and DEG determined in samples used for training in each run, respectively. Both sets of biomarker genes showed strong predictive power (*R* ^2^ = 0.88 and *R* ^2^ = 0.75, respectively). The 17 biomarker genes were found among genes statistically associated with calf BW in the 13 samples used for model training in each run, meaning these genes changed in expression according to calf BW. However, it is noteworthy that the biomarkers found among the gestational genes were also effective in predicting calf BW. These gestational genes were identified from the integration of four studies and change according to the gestational age of the fetus. Consequently, these genes may be more robust in predicting calf BW. These biomarker genes increase in expression during the fetal period, slightly decrease at day 65, and then strongly increase at day 105 (Figure 8). A commonly identified biomarker gene was *IRF7*, which is a key player in the innate immune response to viral infections [56] by serving as a master regulator of type 1 interferon signaling once activated [57]. Interestingly, the prediction of the large calf sample (72.5 kg) was particularly accurate when using the gestational genes (Table 3), reinforcing their potential value for early identification of abnormal fetal growth patterns.

These findings highlight the predictive potential of maternal blood transcriptomics as a noninvasive tool for identifying fetal growth trajectories before birth. The identification of potential biomarkers for calf BW has important implications for precision livestock breeding, allowing early identification of calves at risk for growth restrictions or excessive BW, both of which are associated with neonatal and perinatal complications. In addition, BW prediction offers producers the opportunity to adjust feeding strategies to optimize fetal growth [58], plan interventions for calving, or increase surveillance at parturition to reduce veterinary costs and improve animal welfare. Ultimately, we now have the potential to use supervised machine‐learning algorithms to identify transcript/protein biomarkers to better understand reproductive outcomes in cattle, as well as predict them [59].

While fetal ultrasound measurements have been shown to be strongly correlated with gestational age in cattle [60], their correlation with BW has not been successfully demonstrated. One study assessing fetal ultrasound measurements on day 55 of gestation reported no correlation between either BPD or TD and the size of the fetus that was retrieved from the uterus 1 day later [8]. This is consistent with the findings in the current study and highlights the challenge of finding accurate predictors of calf BW. Fetal growth is highly dynamic and is influenced by many variables. A single ultrasound measurement during early gestation may not fully capture the complexity of the biological and environmental factors that determine BW. Our study suggests that the molecular signatures that can be detected in maternal circulation offer a more robust, reliable alternative to fetal ultrasound measurements, capturing dynamic physiological conditions rather than a single, highly variable snapshot of current anatomical measurements.

### 4.1 Study Limitations

Although the outputs from this study suggest a novel, molecular‐based, minimally invasive approach to predict calf characteristics early in gestation in cattle, several limitations should be considered when interpreting these results. First, the identification of blood genes changing over the course of gestation was performed using a bioinformatic approach that integrated data from four different studies. Ideally, the samples should have been collected from the same animals from the moment of embryo transfer and at various time points during gestation, following a longitudinal time‐course experiment. Nonetheless, we used well‐known bioinformatic methods (Combat and RUVg) to remove technical effects caused by differences between studies and to normalize the data, which was analyzed by maSigPro, a method that supports cross‐sectional time course studies. Moreover, using data from different sources could actually strengthen the results, as the findings are not confined to the biological characteristics of a single group of animals. Second, the selection of biomarker genes was based on a relatively small sample size (20 samples). Therefore, we employed resampling strategies and permutation testing, which are widely regarded as appropriate methods to mitigate bias and evaluate the robustness of predictive models with limited sample sizes [61]. Finally, the results presented are derived from mathematical models applied to transcriptomic data measured through RNA sequencing. Validating these candidate biomarker genes in independent populations could strengthen their predictive potential. Despite these limitations, our study supports the role of maternal blood as a sensor of fetal development and growth in cattle, paving the way for larger future studies that may ultimately optimize pregnancy management to ensure a healthy calf, thereby positively influencing animal welfare.

## 5 Conclusion

In conclusion, this study highlights the potential of analyzing transcriptomic data to determine molecular signatures in the blood that underlie the complexity of pregnancy in the cow. Although integrating transcriptomic data is challenging due to inherent variations among studies, this can be overcome if the bioinformatic pipeline is purpose‐built with due consideration of the biological meaning of the data. High‐throughput omics technologies, coupled with machine‐learning algorithms in the analysis of such biological data, now offer an opportunity to bring ‘precision medicine’ from bench to cow‐side based on their predicted response through the diagnosis of normal pregnancy progression and fetal pathologies via the maternal blood molecular profile. It remains unclear whether these transcriptomic markers in maternal blood are directly influenced by the fetus (and, if so, the mechanism involved), or if they merely reflect broader maternal physiological factors that independently affect calf BW. Future investigations and validations can pinpoint blood changes related to fetal growth or fetal characteristics that could be used to track fetal development or even predict calf health.

## Author Contributions

L.T.: formal analysis; investigation; writing – original draft preparation; writing – review and editing. P.G.H.: formal analysis. M.D.: Formal analysis. D.D.R.: formal analysis. E.M.M.: resources. M.M.: Resources. A.D.C.: resources. S.T.B.: conceptualization, project administration; supervision; writing – review and editing. P.L.: conceptualization, project administration; supervision; writing – review and editing. M.B.R.: conceptualization; formal analysis; project administration; supervision; writing – original draft preparation; writing – review and editing.

## Conflicts of Interest

The authors declare no conflicts of interest.

## Supporting information


**Table S1:** fsb271201‐sup‐0001‐TableS1.xlsx.

**Table S2:** fsb271201‐sup‐0002‐TableS2.xlsx.

**Table S3:** fsb271201‐sup‐0003‐TableS3.xlsx.

**Table S4:** fsb271201‐sup‐0004‐TableS4.xlsx.

**Table S5:** fsb271201‐sup‐0005‐TableS5.xlsx.

**Data S6:** fsb271201‐sup‐0006‐TableS6.xlsx.

## Data Availability

The data that support the findings of this study are openly available in NCBI's Gene Expression Omnibus and are accessible through GEO accession numbers GSE210665, GSE230294, GSE179946, and GSE303819.
